# Prognostic Utility of Oculomotor Assessments in Determining Return-to-Learn Time in Acutely Concussed College Student-Athletes: A Pilot Study

**DOI:** 10.1089/neur.2023.0027

**Published:** 2023-08-11

**Authors:** Maya Vanderhorst, Alex Rawlings, Megan Germansky, Ayana Chodak, Amanda Krohn, Melissa Wilson, Robert Bauman, Benjamin Emke, Robert Parody, Zachary W. Bevilacqua

**Affiliations:** ^1^ Department of Public Policy, Rochester Institute of Technology, Rochester, New York, USA.; ^2^ Department of Exercise Science, Rochester Institute of Technology, Rochester, New York, USA.; ^3^ Department of Athletics, Rochester Institute of Technology, Rochester, New York, USA.; ^4^ Department of Mathematics, Rochester Institute of Technology, Rochester, New York, USA.

**Keywords:** athletic trainer, college, concussion, RTL, return to learn, student-athlete

## Abstract

We sought to discover which oculomotor test (King-Devick [KD], near point of convergence [NPC], and accommodative facility [AF]) would best produce a prognostic model for an RTL time frame. An observational cohort design was used to longitudinally track division I and III student-athletes with concussion at a private university in New York State. Measurements included pre-RTL oculomotor testing (NPC, KD, and AF), along with daily text messages and phone calls. Participants were considered returned-to-learn once they had returned to baseline symptoms and had attended 2 days of classes. Our data promote KD score and class attendance as the best-fit prognostic model, with every second accrued on the KD test equating to 5.29 h of RTL time. Further, attending class throughout recovery, versus not, shortened RTL time by a mean 170.50 h, or 7.1 days. Five variables produced a significant attenuating association with concussion symptoms: time post-injury (*p* = 0.01); caffeine (*p* = 0.05); alcohol (*p* = 0.01); music (*p* = 0.01); and physical activity (*p* = 0.01). Three variables produced a significant worsening association with concussion symptoms: screen time (*p* = 0.05); music (*p* = 0.01); and class attendance (*p* = 0.01). The findings present a preliminary evidence-based model to prognosticate RTL time. To our knowledge, this is the second longitudinal study, and the first overall, to present objective data for guiding and prognosticating RTL, respectively. Correspondingly, these data should assist clinicians with objectively steering RTL in-clinic.

## Introduction

The return-to-learn (RTL) arm of concussion management is comparatively underdeveloped to standardized return-to-play protocols,^1,2^ particularly for college students. For instance, a return to symptom-free exercise is objectively guided by heart-rate data^3^ and can even prognosticate a person's recovery time.^4^ Sadly, no commensurate measures are available for RTL. Instead, the National Collegiate Athletic Association's RTL protocol broadly states: “A student-athlete who has suffered a concussion will return to classroom/studying only as tolerated with modification,” leaving clinicians with limited ability to steer recovery.^5^ Resultantly, available systematic review suggests that clinicians support RTL by prescribing a temporary absence from school and assess for vestibulo-ocular deficits that may translate to classroom difficulties.^6^ Considering that 29%^7^ and 53%^8^ of sample concussed college students display visual and oculomotor symptomology, ocular screening may represent a viable tool for shaping an RTL plan of care.

The associations between oculomotor deficits and academic disruption are repeatedly observed. Visual symptoms (i.e., photophobia, blurred vision) were significantly associated with academic difficulty in 5- to 18-year-olds (odds ratio [OR], 2.17; 95% confidence interval [CI]: 1.02, 4.62) and remained associated in those with prolonged symptoms (OR, 3.15; 95% CI: 1.06, 9.38).^9^ Corwin and colleagues found similar outcomes, given that a higher percentage of students who initially presented with oculomotor abnormalities required school accommodations and remained symptomatic longer than 4 weeks.^10^ Recently, near point of convergence (NPC) scores were shown to positively correlate with reading rate in concussed student-athletes, ages 14–22.^11^ These data are logical, in that academic tasks demand prolonged, repetitive, and accurate visual and oculomotor function. Moreover, school activities (e.g., reading, viewing) closely mimic smooth pursuit, saccadic, and accommodative assessments, further underpinning the relevance of an oculomotor screening in-clinic.

When determining a student's “readiness” to RTL, clinicians must largely rest their judgement on subjective symptom reports. This is troubling, given that students are known to purposefully minimize the quantity and severity of their symptoms, in hopes of returning to sport, school, and social activities prematurely.^12–18^ To bypass this, and progress clinical management for RTL, objective measures must be created. The inclusion of objective oculomotor testing to a future set of RTL guidelines would provide easily accessible, applicable tools to anticipate a student's academic recovery; therefore, we sought to discover which oculomotor test (King-Devick [KD], NPC, or accommodative facility [AF]) would best produce a prognostic model for a RTL time frame. These tests were chosen for their quantifiable outcome measures (i.e., time, distance, and errors) and striking resemblance to eye movements during routine academic tasks.

## Methods

### Participants

Fourteen student-athletes from the Rochester Institute of Technology were longitudinally tracked. To be considered for the study, participants must: 1) be ≤7 days post-injury, 2) be enrolled as a full-time student (undergraduate ≥12 credits, graduate ≥9 credits), 3) be between 18 and 26 years old, and 4) have not attended class since their injury. Students were ineligible if they reported: 1) a separate head, neck, or face injury in the 6 months preceding the study, 2) history of vestibular, ocular, or neurological dysfunction unrelated to their current concussion, 3) were pregnant, or 4) unable to correctly answer all orientation questions on the Sport Concussion Assessment Tool 5.^19^ All participants were diagnosed by a member of the Rochester Institute of Technology Athletic Training Staff, using the 5th consensus definition of concussion.^20^ Full demographic details are seen in Table 1.

**Table 1.** Participant Demographics

	Female	Male	*p* value
No.	7	7	
Age, years	19.75 ± 1.04	20.66 ± 1.86	0.38
Weight (lb)	148.13 ± 26.58	164.17 ± 15.94	0.18
Height (in)	65.00 ± 2.83	70.83 ± 2.31	0.0015
			
Hx of physician treatment for:			
Headaches or migraines	Yes, 3	Yes, 0	
Sleep disorder	Yes, 0	Yes, 0	
Substance or alcohol abuse	Yes, 0	Yes, 0	
Anxiety or depression	Yes, 3	Yes, 1	
Diagnosed with ADD/ADHD	Yes, 0	Yes, 0	
			
Current injury			
No. of previous concussions	0.66 ± 0.71	0.86 ± 1.46	0.76
Days Until RTL Completed	13 ± 6 (*med: 12, min: 7, max: 22*)	16 ± 6 (*med: 13, min: 9, max: 28*)	0.38
			
Pre-injury symptom levels			
Headache	0.75 ± 1.04	0.29 ± 0.76	0.35
Fatigue	1.00 ± 2.07	0.71 ± 0.95	0.74
Dizziness	0.13 ± 0.35	0	0.37
Difficulty concentrating	0.63 ± 1.19	0.57 ± 1.33	0.93
Anxiety	0.86 ± 1.25	0.57 ± 0.96	0.61
Sensitivity to light	0	0	
Total pre-injury symptom score	3.36 ± 3.85	2.14 ± 2.41	0.48
			
Academic information			
Undergraduates	7	7	
Academic majors represented	Biomedical Sciences; New Media Design; Mechanical Engineering; Civil Engineering Technology; Packaging Science; Criminal Justice; Physician Assistant; Sign Language Interpreting	Undecided; Business; Biomedical Engineering; Computing Security; Management Information Systems; Accounting	
Semesters completed, bachelors	2.75 ± 2.31	3.43 ± 2.07	0.56
Receiving accommodation before concussion	Yes, 0	Yes, 0	
No. of credits online	2.00 ± 2.14	4.00 ± 3.61	0.21
No. of credits between 8:00 am and 12:00 pm	6.63 ± 4.07	5.29 ± 2.43	0.46
No. of credits between 12:00 pm and 4:00 pm	4.75 ± 3.58	4.14 ± 3.18	0.73
No. of credits between 4:00 pm and 8:00 pm	2.63 ± 3.02	1.00 ± 1.41	0.22
			
Pre-RTL oculomotor scores			
AF	68.00 ± 16.46	77.14 ± 19.34	0.34
NPC	12.63 ± 3.80	9.33 ± 3.92	0.12
KD	60.76 ± 9.21	52.93 ± 12.57	0.20

Values are expressed as means ± standard deviation.

Hx, medical history; ADD, attention deficit disorder; ADHD, attention deficit/hyperactivity disorder; MVA, motor vehicle accident; AF, accommodative facility; NPC, near point of convergence; KD, King-Devick.

### Procedure

After the collection of demographic data, participants completed a battery of oculomotor assessments (NPC, KD, and AF), given in a randomized order by a member of the research team. Consistency between administrators was anticipated, with these tests possessing high inter-rater reliability (NPC = 0.95,^21^ KD = 0.95,^22^ AF = 0.89^23^). Participants' scores represented their pre-RTL values and were taken before the student having returned to class. Next, participants were fitted with an ActiGraph wristwatch^24^ (ActiGraph wGT3X-BT) and instructed to wear the watch at all times. Finally, participants were instructed to respond promptly to the text messages and phone calls they would receive daily. The ActiGraph watch and phone calls provided the research team with behavioral data (participation in physical activity, sleep, hydration, music listened to, etc.), which have been shown to influence symptom levels and RTL time frame by extension.^25^ In turn, these and any other hypothesized covariates were monitored longitudinally to minimize statistical error of our model.

Participants remained in the study until they reached completion criteria, or failed to reach completion criteria in 30 days. Completion criteria were consistent with previous work^25^ and were defined as a 2-day period of class attendance (online or in-person) at or below pre-injury symptom levels. Participants were removed from the study if they: 1) answered <80% of texts or phone calls, 2) voluntarily withdrew, or 3) were found to be not wearing the ActiGraph watch. The Rochester Institute of Technology Institutional Review Board approved this study (#02062821).

### Materials

#### Accommodative facility

**FIG. 1.**
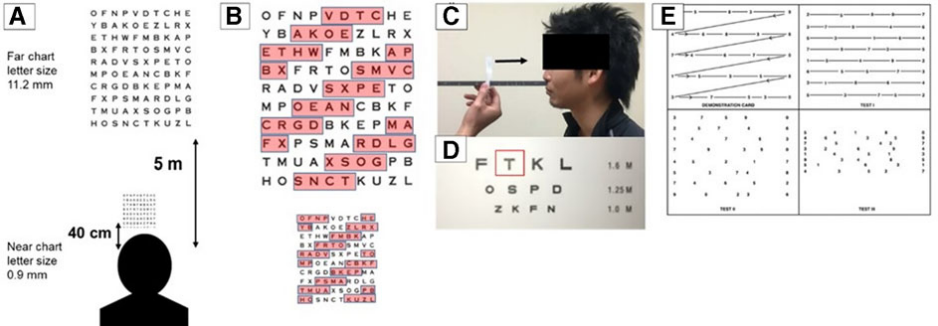
Oculomotor tests. (**A**) AF exam setup. (**B**) Identical charts (Hart Charts) used during the AF, showing the letters (highlighted) a participant will recite as they alternate back and forth between the near chart and far chart. (**C**) NPC exam setup, with arrow indicating the target's gradual motion toward the participant's eyes. (**D**) NPC target card; participants are told to keep the letter “T” in focus as long as possible. (**E**) KD exam cards, with guidelines indicating the left-to-right and top-to-bottom directions of reading. AF, accommodative facility; KD, King-Devick; NPC, near point of convergence.

AF is commonly used in ophthalmic practice to assess binocular accommodation, but is new to concussion research. This test requires patients to reciprocally transition sight between a distant (5 m) and near (40 cm) Hart Chart displaying an identical sequence of characters (letters and numbers; Fig. 1A). This test is ideal to mimic the transitioning of sight from a distant projector screen to a student's laptop screen, performed commonly in the classroom. Patients recite characters in the same fashion as the KD (left to right, top to bottom), but transition sight to the opposing chart after every fourth character. Figure 1B shows which characters are read from which Hart Chart (red highlight; highlighting not present during testing). Though parameters for the near chart are agreed upon (40 cm distance),^26–29^ there are varying recommendations for the distant chart (3–6 m).^27–30^ We chose a distance of 5 m to stay within the published range.

Participants were instructed to read as much of the chart, as fast and accurately as possible, in 1 minute. Whereas a time limit is atypical for the AF test, we sought to mimic the fast-paced nature of a college classroom, by replicating the brief period in which course materials are displayed. Because AF is novel to concussion investigations, we took certain liberties in how a participant's score should be determined. In turn, we decided to assign a score based on the total number of accurate characters read in 1 minute. The Hart Chart is a 10 × 10 grid; therefore, reading the entire chart would result in a score of 100. Errors (i.e., skipped or misread characters) were subtracted from a participant's total at the end of the minute (e.g., 77 characters read, 4 errors recorded; final score = 73). Errors were recorded by the tester.

#### Near point of convergence

NPC was assessed with a Gulden Near Point Rule,^31^ utilizing a recommended^32–34^ target speed of 1–2 cm per second (Fig. 1C). Participants were instructed to focus on the letter “T” in the top row of the accommodation card (Fig. 1D) and report when diplopia would occur. Convergence distance was determined when the participant reported diplopia, or ocular abduction was observed by the tester. Three trials were conducted and a mean calculated. Because NPC assesses the ability to maintain focus on an object in close proximity to the face, it resembles a student holding a gaze on their laptop screen or notebook.

#### King-Devick

KD was assessed using a tablet held by the participant and the KD sideline application.^35^ Participants were told to read aloud, from left to right, top to bottom, as fast and as accurately as possible (Fig. 1E). A demonstration card was used to acclimate participants, followed by three test cards. Total time (in seconds) to read the test cards was internally recorded by the application, with the number of errors recorded by the tester. KD directly imitates the unavoidable school task of reading, given the likeness between oculomotor patterns and comprehension of written characters.

### Text messages and phone calls

Text messages were sent to participants in a repeated measures fashion four times per day (9:00 am and 1:00, 5:00, and 9:00 pm) and were used to determine resumption of baseline symptom levels. Texts inquired about six symptoms: headache, dizziness, difficulty concentrating, fatigue, anxiety, and sensitivity to light. These symptoms were chosen based on their high prevalence in collegiate cohorts^7^ or the population in general.^36^ Phone calls were made daily at 6:00 pm by a member of the research team and gathered data on student behaviors. Because the studied institution continued to offer hybrid or online options to combat COVID-19, two additional questions were added to the phone script, versus its predecessor^25^: 1) “Which classes did you attend online?”; 2) “How did you participate in these classes? (watched and listened, just watched, just listened) + (in real time, utilized recording later).” For expansive details on text message and phone call scripts, please refer to Supplemental Materials S1.

### Statistical analysis

#### Prognostic model

To begin, we conducted a mixed-effects regression model that included the six symptoms as outcome variables and 10 independent behavior variables: post-injury time, step count, sleep, water, caffeine, alcohol, screen time, music, class attendance, and physical activity. Independent variables that produced statistically significant estimate values were then incorporated into the prognostic model as covariates. An Akaike information criterion (AIC) approach, through step-wise regression, was used to create a model to best predict RTL time. This approach to generating an RTL model is preferred over *p* values, given that some correlation between test variables (i.e., oculomotor scores) is anticipated.

## Results

### Confounding behaviors

Five variables produced a significant attenuating association with concussion symptoms: time post-injury (*p* = 0.01); caffeine (*p* = 0.05); alcohol (*p* = 0.01); music (*p* = 0.01); and physical activity (*p* = 0.01). Three variables produced a significant worsening association with concussion symptoms: screen time (*p* = 0.05); music (*p* = 0.01); and class attendance (*p* = 0.01).

### Prognostic model

The AIC model to predict time until full RTL included both KD and class attendance. Specifically, every second accrued during a KD test was equivalent to 5.29 h of time until full RTL. Additionally, class attendance throughout recovery, versus not, reduced time until full RTL by an average 170.50 h, or 7.1 days.

## Discussion

The aim of the current study was to present clinicians with a prognostic model to objectively predict time until full RTL. Our findings provide such a model, which we will discuss here.

### Prognostic model

Our data promote KD score and class attendance as the best-fit prognostic model. To our knowledge, this study is the first to record both KD scores post-concussion and RTL recovery time; therefore, the following comparison to existing literature is largely anecdotal. Considering that mean values for RTL time are greater than median times (mean days: 18.3^25^; median days: 8^37^), we can loosely assume that RTL distribution is positively skewed. KD scores behave similarly (mean seconds: 47.0^38^, 50.0^39^; median seconds: 45.8^40^), directing us to use median values as comparators. Through our prognostic model (i.e., one second = 5.29 h), a median KD score of 45.8 sec would predict a 10-day RTL time. Ten days bisects the period in which roughly 50–75% of college students will have returned-to-learn (i.e., 7–14 days); yet, it closely falls within 48 h of previously published median RTL times (8 days).^37^ We attribute the overall accuracy of our model to the unmistakable similarities between KD testing and class activities.

Regardless of how class is attended (online, in-person), saccadic and smooth pursuit eye movements expectedly occur. In fact, when online classes are attended, it more closely resembles administration of the KD test, in that both stimuli (laptop screen, KD numbers) are delivered through LED screens ∼12–24 inches from the eyes. Fifty-seven percent of our cohort attended at least one online class throughout their recovery, with 27% of classes across all participants attended online. It can be said with some certainty that online or remote learning is a useful tool in the college setting. Students with concussion may benefit from this platform by attending class without burdening themselves with cross-campus travel. Additionally, student-athletes are routinely taken away from the classroom because of travel, to which online attendance can rectify.

Universities stress continuity of instruction when students are unable to physically attend class (e.g., concussion, Covid-19, etc.), and though both faculty and students prefer in-person education versus remote,^51^ there is no question that virtual education has augmented its presence. Therefore, to supplement this model, we recommend that clinicians inquire about their patient's online course load and recommend using recorded lectures whenever possible. Understanding the patient's online course load, in combination with their KD scores, allows a clinician to educate a patient on the difficulty they may have re-engaging with their classes (i.e., poor KD score and several online courses equals greater difficulty).

Recorded lectures provide a solution, whereby students are encouraged to watch class, but have the option to periodically rest ocular muscles. Moreover, faculty indicate that recording their lectures is feasible to do,^1^ with 53% of faculty believing that audio-recorded lectures should be provided to concussed students.^41^ Last, our data associate class attendance with increased difficulty concentrating and anxiety. Watching recorded lectures may alleviate both of these, given that students have the ability to replay information at their own pace.

### Limitations

Although the present study adds novel evidence to RTL, it is not without limitations. Most notable are our sample size and use of a single university, which limits the range of courses and students (major, difficulty, and level of study) we can present. The small sample size also limits the generalizability of the study, but can serve as a foundation for a follow-up multi-center investigation. To keep athletic trainers unbiased in their practice, a participant's medical recommendations or provider referrals (e.g., vestibular therapy, medication, and RTP progression) were not tracked, but could have potentially influenced participant behavior. Last, we defined recovery as a 2-day period of class attendance at or below pre-injury symptom levels; however, we do not tease out whether a student attended all scheduled classes for that day or just a portion. This could mean that a student has not fully resumed their entire course load, but is considered returned-to-learn.

Our thought behind this decision was in response to the several reasons why a student may not attend all classes for the day. We have already mentioned that student-athletes are pulled away from class because of travel or team activities. Additionally, some classes may be canceled because of weather, instructor discretion, or do not meet weekly. Therefore, we chose to simplify what constitutes class attendance, for fear of overestimating RTL time because of class absences outside of the student's control.

## Conclusion

Our study presents a novel and objective prognostic model for RTL that aligns well with previously published RTL times. These pilot data serve as justification for future investigations to validate this model through a multi-center effort. A prognostic model for RTL will provide clinicians with a tool to guide their concussed college-aged patients, while also allowing students to anticipate when they can resume symptom-free academic activity.

## Supplementary Material

Supplemental data

## Abbreviations Used

AF: accommodative facility
AIC: Akaike information criterion
CI: confidence interval
KD: King-Devick
NPC: near point of convergence
OR: odds ratio
RTL: return-to-learn
