# Supplementary material for: Prognostic Utility of Oculomotor Assessments in Determining Return-to-Learn Time in Acutely Concussed College Student-Athletes: A Pilot Study
Source: Neurotrauma Rep. 2023 Aug 11;4(1):515–21. doi: 10.1089/neur.2023.0027 (PMC10457635; doi:10.1089/neur.2023.0027)
Supplement: Supplemental data [file Supp_DataS1.docx]

Text Messages

The text messages will gather the severity of six symptoms, and will read as follows:

*Please rate your current symptoms on a 0-10 scale (0 = none, 10 = most severe).*

1. *Headache*
2. *Dizziness*
3. *Difficulty Concentrating*
4. *Fatigue*
5. *Anxiety*
6. *Sensitivity to Light*

Patients will be told to respond to the text messages in the following format (see participant instructions document):

*A. 4 B. 2 C. 8 D. 0 E. 1 F. 6*

**Daily Phone Call Questions Script**

“All questions are referencing the period of time between your last phone call, till now”

1. “How many 8 oz servings of water did you drink?”
2. “How much caffeine did you drink? For example, soft drinks coffee or tea enter. As a reference a typical bottled soft drink is 16 oz?”
3. “How much alcohol did you drink? As a reference a typical can of beer is 12 oz?”
4. “What classes did you attend?”
5. ***IF THEY ATTENDED CLASSES***
   1. “Did you feel better, worse, or no change during those classes? For example, you can say, in math I felt worse, in art I felt no change”.
   2. “Which of these classes were completed online today?”
      1. “How did you participate in this class and why? For example, you can say I watched and listened to the lecture in real time, I only listened to the lecture in real time, I utilized the recording of the lecture and watched or just listened.
6. “How much screen time have you engaged in? For example, TV, texting, or computer”
7. “How much time have you spent listening to music outside of class?”
8. “Have you performed any physical activity?”
9. ***IF YES*** “What was the type and duration of physical activity you performed? For example, you could say, I rode a stationary bike for 30 minutes”.
